# In vivo transcriptional targeting into the retinal vasculature using recombinant baculovirus carrying the human flt-1 promoter

**DOI:** 10.1186/1743-422X-4-88

**Published:** 2007-09-18

**Authors:** Agustín Luz-Madrigal, Carmen Clapp, Jorge Aranda, Luis Vaca

**Affiliations:** 1 Departamento de Biología Celular, Instituto de Fisiología Celular, Universidad Nacional Autónoma de México (UNAM), Ciudad Universitaria, México D.F. 04510, México; 2 Instituto de Neurobiología, UNAM-Juriquilla, Querétaro, Qro México, 76001, México

## Abstract

**Background:**

Endothelial cells are a target for gene therapy because they are implicated in a number of vascular diseases. Recombinant baculovirus have emerged as novel gene delivery vectors. However, there is no information available concerning the use of endothelial-specific promoters in the context of the baculovirus genome. In the present study, we have generated a recombinant baculovirus containing the human flt-1 promoter (BacFLT-GFP) driving the expression of the green fluorescent protein. Transcriptional gene targeting was analyzed *in vitro *in different mammalian cell lines and *in vivo *in adult rat retinal vasculature.

**Results:**

BacFLT-GFP evoked the highest levels of expression in the endothelial cell line BUVEC-E6E7-1, similar to those reached by recombinant baculovirus carrying the CMV promoter (112% relative to BacCMV-GFP, *n *= 4). Interestingly, BacFLT-GFP directed high levels of expression in rat glioma C6 and in human glioblastoma CH235 cells (34.78% and 47.86% relative to BacCMV-GFP, respectively). Histone deacetylase inhibitors such as butyrate or trichostatin A enhanced the transcriptional activity of both BacCMV-GFP and BacFLT-GFP. Thus, in this study histone deacetylation appears to be a central mechanism for the silencing of baculovirus, independently of the promoter utilized. *In vivo *transcriptional targeting was demonstrated in adult rat retinal vasculature by intravitreal delivery of BacFLT-GFP and immunohistochemical staining with von Willebrand factor (vWF). Analysis by fluorescence microscopy and deconvolved three-dimensional confocal microscopy of retinal whole mounts obtained after 3 days of baculovirus injection showed that most GFP-expressing cells localized to the inner limiting membrane (ILM) and ganglion cell layer (GCL) and colocalize with vWF (70%, *n *= 10) in blood vessels, confirming the endothelial phenotype of the transduced cells.

**Conclusion:**

Taken together, our results indicate that the restricted expression in endothelial cells mediated by the flt-1 promoter is not affected by the context of the baculovirus genome and demonstrate the potential of using recombinant baculovirus for transcriptional targeted gene expression into the eye vasculature.

## Background

Local delivery of genes to vascular wall is a promising approach for the treatment of a number of vascular disorders [1]. As a target organ for gene transfer, the vasculature has several unique features such as a large surface area and easy accessibility. The architecture of the normal vessel wall is relatively simple consisting of three main cell types (endothelial cells, smooth muscle cells, and fibroblasts) and the transgene products may be secreted locally to achieve an autocrine-paracrine effect or into the bloodstream for a systemic effect. Within the vasculature, endothelial cells are the main target for gene therapy because they are closely related with disease process such as inflammation, atherosclerosis, systemic and pulmonary hypertension, cerebrovascular disease, and in angiogenesis-related disorders [1]. Moreover, tumor angiogenesis is crucial for the progression and metastasis of cancer [2]. Therefore, tumor vascular targeting therapy could represent an effective therapeutic strategy to suppress both primary tumor growth and tumor metastasis [2].

Viral vectors have been used extensively in vascular gene transfer; adenoviral vectors being the most commonly used system [3]. Other vector systems include adeno-associated virus (AAV) and lentiviral vectors [4]. Although these vectors have demonstrated the transfer of genetic material for its expression in endothelial cells, the main limitations are associated with inflammatory reactions due to the pre-existing immunity to human virus [4,5]. To address this problem, the use of recombinant viruses of non-human origin as gene therapy vectors has been suggested [6].

Recently, recombinant baculovirus derived mainly from *Autographa californica *multiple nuclear polyhedrosis virus (AcMNPV) have emerged as a novel and safer system to transfer genes for its expression into a wide variety of mammalian cells [7]. Since the first studies made by two different groups, showing the ability of baculovirus to transfer genes in mammalian cells derived from hepatic origin [8,9], the list of mammalian cells susceptible to transduction by recombinant baculovirus has increased in the last few years [7].

Transcriptional targeting using cellular tissue-specific regulatory sequences has been demonstrated as a powerful strategy to restrict gene expression to a particular cell type in various tissues, including liver, smooth muscle and heart [10,11]. Moreover, utilization of tumor/tissue-specific promoters can reduce toxicity, increase safety, and improve the therapeutic index [12,13].

The human transmembrane *fms*-like tyrosine kinase (Flt-1) is one of the receptors for vascular endothelial growth factor (VEGF) [14]. Flt-1 is expressed specifically in endothelium and is likely to play a role in tumor angiogenesis and embryonic vascularization [15]. Morishita *et al*., demonstrated that a 1-kb DNA fragment of the 5'-flanking region of human *flt-1 *gene (region from -748 to +284 bp) is involved in endothelial-specific gene expression [16]. So far, there is no information available concerning the use of endothelial-specific promoters in the context of the baculovirus genome. Furthermore, only two reports show to this date *in vivo *transcriptional gene targeting by recombinant baculovirus.

In this study, we produced a recombinant baculovirus (BacFLT-GFP) containing the human flt-1 promoter driving the expression of the green fluorescent protein (GFP) and evaluated the maintenance of endothelial-specific gene expression after *in vitro *transduction of different mammalian cell lines. We also demonstrated *in vivo *transcriptional targeting into the rat retinal vasculature by immunoflurescence staining after intravitreal delivery of BacFLT-GFP. Three-dimensional (3-D) confocal reconstruction studies of retinas from animals injected with BacFLT-GFP showed for the first time the selective targeting to blood vessels of a baculovirus vector.

## Results

### Transduction susceptibility mediated by recombinant baculovirus in mammalian cells

**Figure 1:**
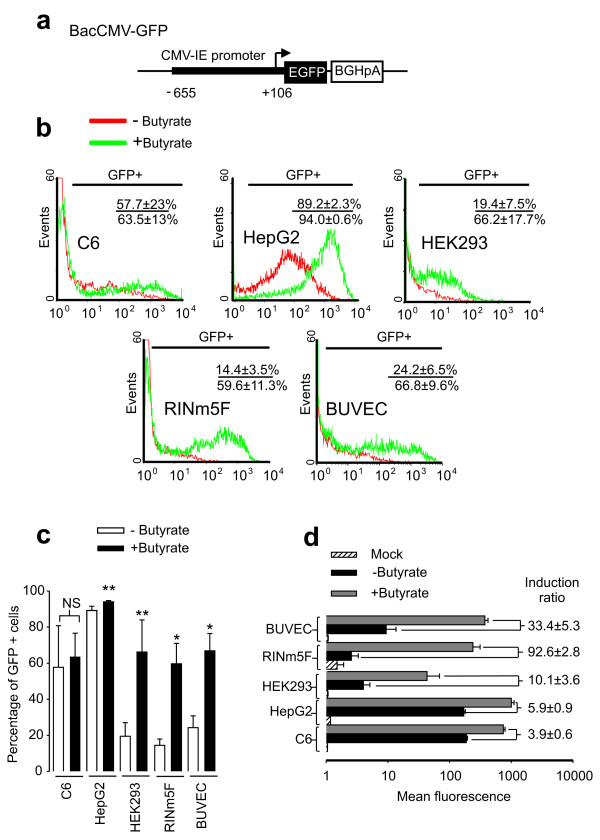
**Transduction susceptibility mediated by BacCMV-GFP in mammalian cells**. 10^5 ^cells were transduced at a multiplicity of infection (MOI) of 100 with BacCMV-GFP. (a) Drawing showing the cassette structure of BacCMV-GFP. Abbreviations: CMV, cytomegalovirus immediately-early promoter/enhancer (nucleotides -655 to +106); EGFP, enhanced green-fluorescent protein; BGHpA, bovine growth hormone poly adenylation sequence. (b) Representative histograms obtained by flow cytometry after 48 h post-transduction. The percentage of GFP+ cells is reported in the insets, and was calculated by subtracting the background obtained with mock transduced cells (see Methods). Numbers in the inset refer to the percentage of GFP+ cells ± SD without (above) or with (below) treatment with butyrate. (c) Percentage of GFP+ cells determined by FACS analysis in either the presence or absence of butyrate. *P < 0.01, **P < 0.05, ***P = 0.7244 versus cells non-treated with butyrate. NS = non significant. d) Levels of expression of GFP and induction ration (indicated on the right) as determined by the mean fluorescence intensity in the presence or absence of butyrate. Values are means ± SD of four independent experiments.

To compare the selectivity and the levels of expression mediated by the endothelial specific baculovirus (BacFLT-GFP), we generated a recombinant baculovirus containing a 761-bp DNA fragment of the cytomegalovirus (CMV) promoter driving the expression of GFP (Figure 1a). This promoter was selected because it drives high levels of expression into mammalian cells from different tissues. We included the immortalized bovine umbilical vein endothelial cell line BUVEC-E6E7-1, which retains endothelial cell characteristics and has been utilized to investigate the action of regulatory factors of vascular endothelium [17]. The cells were transduced at a multiplicity of infection (MOI) of 100 in the presence or absence of 5 mM butyrate, a histone deacetylase inhibitor. The percentage of GFP positive (GFP+) cells and the levels of expression, represented by the mean fluorescence intensity (MFI), were analyzed 48 h after transduction by flow cytometry. In all cases mock-transduced cells (cells treated with medium alone and butyrate) were used as control for background fluorescence. In the experiments performed without the addition of butyrate (Figure 1b,c) the hepatocarcinoma cell line HepG2 was the most susceptible to transduction, with 89.2 ± 2.3% GFP positive cells, followed by the rat glioma cell line C6, 57.7 ± 23%; the bovine umbilical vein endothelial cell line BUVEC-E6E7-1, 24.2% ± 6.5%; the human embryonic kidney cell line HEK-293, 19.4 ± 7.5% and the rat insulinoma cell line RIN-m5F, 14.4 ± 3.5%.

Modification of the accessibility of the transcription machinery to gene promoters is one of the underlying mechanisms regulating gene expression in mammalian cells [18]. Although several mechanisms are involved in the regulation of this process, the status of the histone acetylation/deacetylation and DNA methylation/demethylation seems to be of major importance [18,19]. Previous reports have suggested that epigenetic regulation seems to influence the transduction efficiency mediated by recombinant baculovirus in mammalian cells [20,21]. Therefore, we treated the cells with 5 mM butyrate, which has been used to reactivate transgene expression by inhibiting the chromatin-remodeling of histone deacetylases (HDAC), which results in an increase of transcriptional activity of the promoter [22]. Treatment with butyrate (Figure 1b,c) dramatically reactivated the expression in almost all cell lines, except in C6 in which there was no significant difference from control (63.5 ± 13% and 57.7 ± 23% of GFP+ cells for control and butyrate, respectively; *P *= 0.7244, *n *= 4). In agreement to previous reports [20,21], levels of expression evaluated by the MFI were increased by addition of butyrate (Figure 1d). Particularly, the cell line RIN-m5F was the most susceptible to reactivation by butyrate (92.6 ± 2.8-Fold), followed by BUVEC-E6E7-1, and HEK-293 cells (33.4 ± 5.3 and 10.1 ± 3.6-Fold, respectively). In an attempt to reactivate gene expression by DNA methylation inhibitors, we treated the cells with 5'aza-2'deoxycytidine (5'aza-C) [23], however there was not a significant difference between the levels of expression reached by control untreated cells and treated with 30 μM of 5'aza-C (data not shown). These results suggest that in these cell lines including BUVEC-E6E7-1 cells, there is a strong repression of the transgene expression most likely due to histone deacetylation. In contrast, HepG2 and C6 were less susceptible to reactivation (5.9 ± 0.9 and 3.9 ± 0.6-fold, respectively) indicating that in these cell lines histone deacetylation does not play a critical role in gene silencing the baculovirus genome.

### Baculovirus containing the human Flt-1 promoter drives endothelial-specific gene expression *in vitro*

**Figure 2:**
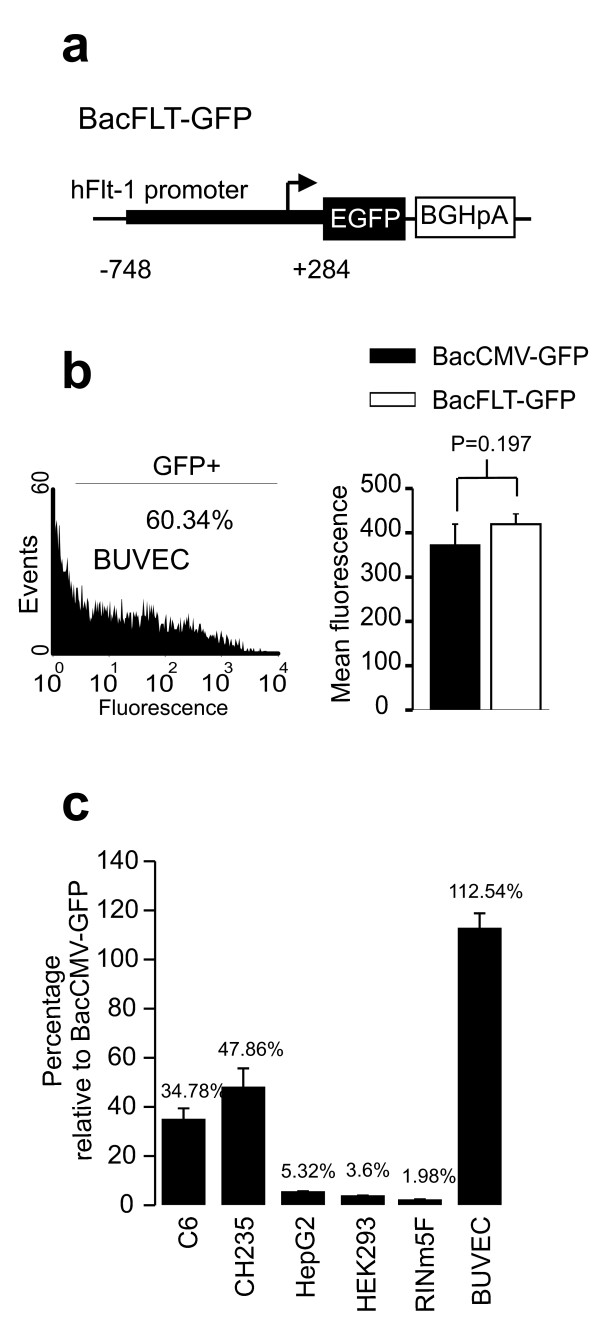
**Specificity and levels of gene expression mediated by BacFLT-GFP**. (a) Drawing showing the cassette structure of BacFLT-GFP. Abbreviations: hFlt-1, promoter sequence of the human *flt-1 *gene (nucleotides -748 to +284 bp); EGFP, enhanced green-fluorescent protein; BGHpA, bovine growth hormone poly adenylation sequence. (b) (Left panel), Representative histogram obtained by flow cytometry 48 h after transduction of BUVEC-E6E7-1 cells with 100 MOI of BacFLT-GFP and 5 mM of butyrate. The percentage of GFP+ cells is reported in the inset, and was calculated by subtracting the background from mock transduced cells (see Methods). (Right panel) Levels of expression obtained with BacCMV-GFP and BacFLT-GFP in BUVEC-E6E7-1 cells, representative of four independent experiments, mean ± SD. (c) Mean fluorescence intensity (MFI) measured 48 h after transduction with BacFLT-GFP relative to BacCMV-GFP. The percentage of GFP+ cells are indicate above of each bar. Cells were transduced with 100 MOI of BacFLT-GFP or BacCMV-GFP. Data are from four independent experiments ± SD.

We generated a novel recombinant baculovirus with the human flt-1 promoter driving the expression of GFP (BacFLT-GFP, Figure 2a) to test its selectivity and efficiency as a vascular vector for gene therapy.

We first assessed the efficiency of transduction and levels of expression mediated by BacFLT-GFP in the immortalized bovine umbilical vein endothelial cell line BUVEC-E6E7-1 in the presence of 5 mM butyrate. Analysis by flow cytometry 48 h after transduction with BacFLT-GFP (MOI of 100) showed a large number of GFP+ cells (60.34%, *n *= 4), similar to that obtained with BacCMV-GFP (Figure 2b). Moreover, there is not significant difference between the levels of expression reached by BacFLT-GFP and BacCMV-GFP at the same MOI (Figure 2b right panel, *P *= 0.197, *n *= 4). Similar results were obtained by Nicklin *et al*., in which recombinant adenoviral vector containing the same promoter sequence produced levels of expression comparable to those evoked by an adenovirus containing the CMV promoter in human umbilical vein (HUVEC) and human saphenous vein endothelial cells (HSVEC) [24].

We next analyzed levels of expression in non-endothelial cells to evaluate *in vitro *cell specificity (Figure 2c). Since the efficiency of gene transfer differs among cell types, the levels of expression (i.e. MFI) for each cell line were normalized using BacCMV-GFP as positive control.

The highest level of expression with BacFLT-GFP was observed in BUVEC-E6E7-1 cells (112 ± 6.2% relative to BacCMV-GFP, *n *= 4). Importantly, the levels of expression were severely reduced in other non-endothelial cell types including HepG2, HEK293 and RINm5F. For example, GFP expression in HepG2 cells was only 5.32 ± 0.37%, of that obtained with BacCMV-GFP, demonstrating the low activity of flt-1 promoter in non-endothelial cells (Figure 2c). Furthermore, BacFLT-GFP produced high levels of expression in the rat glioma C6 and in the human glioblastoma CH235 cells (34.78 ± 4.6, 47.86 ± 7.84, respectively). In agreement with our results, Flt-1 and KDR (both receptors for VEGF) have been identified on malignant cells from human CNS, breast, prostate cancer and in cell lines derived from these tumors [15,25]. Furthermore, RT-PCR studies of C6 and CH235 cells showed high levels of expression of the Flt-1 receptor mRNA (data not shown).

In summary, these results indicate that the flt-1 promoter retains its transcriptional selectivity in the context of baculovirus genome *in vitro*.

### Histone deacetylase inhibitors reactivate the expression mediated by recombinant |baculovirus containing the human Flt-1 promoter

**Figure 3:**
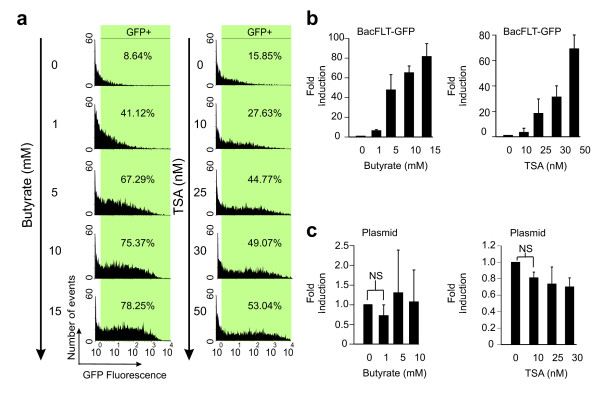
**Effect of histone deacetylase inhibitors in the expression mediated by BacFLT-GFP**. (a) Representative histograms obtained by flow cytometry 48 h after transduction of BUVEC-E6E7-1 cells with 100 MOI of BacFLT-GFP and treated with increasing concentrations (shown in the figure) of butyrate or trichostatin A (TSA). The percentage of GFP+ cells (shown inside green rectangles) is reported in the insets, and was calculated by subtracting the background from mock transduced cells. (b) Fold induction in GFP expression mediated by BacFLT-GFP with increasing concentrations of butyrate or TSA. (c) Fold induction of the expression of GFP in transfected cells with the transfer plasmid pBlueFLT-GFP (see Methods) and treated with increasing concentrations of butyrate or TSA. Results from four independent experiments ± SD. NS = non significant.

To analyze whether HDAC inhibitors can also improve recombinant baculovirus-mediated gene expression under the control of flt-1 promoter, BUVEC-E6E7-1 cells were transduced with BacFLT-GFP at an MOI of 100 in the presence of different concentrations of butyrate or TSA (Figure 3a). The GFP+ cells and MFI were examined by flow cytometry 48 h post-transduction. Treatment with both HDAC inhibitors clearly improved gene expression in terms of GFP+ cells in a dose-dependent manner (Figure 3a). The number of GFP+ cells was increased from 8.64% in untreated control cells to 78.25% in cells treated with 15 mM butyrate. Butyrate inhibits HDAC but also has a number of unrelated effects [22]. To determine whether the inhibition of histone deacetylation was the major contributor to enhanced GFP expression, cells were treated with TSA, which is a more potent and selective inhibitor of deacetylases [26]. TSA significantly enhanced transgene expression in a dose-dependent manner, ranging from 15.85% in untreated control cells to 53.04% GFP+ in cells treated with 50 nM TSA (Figure 3a, right panel). In both cases (butyrate or TSA) the range of fluorescence intensities in the population is spread over approximately three orders of magnitude, suggesting different levels of expression within the cells. Improvement of gene expression after treatment with butyrate or TSA was also evidenced by MFI (Figure 3b). The average level of induction was 81.68-fold and 61.69-fold for 15 mM butyrate and 50 nM TSA, respectively. There were no significant differences in expression at concentrations between 5 and 10 mM of butyrate or 25 and 30 nM of TSA (Figure 3a,b). Higher concentrations of butyrate or TSA administrated for 48 h resulted in toxic effects in terms of viable cell number evaluated by trypan blue exclusion.

We performed transient transfections in BUVEC-E6E7-1 cells using the transfer plasmid pBlueFLT-GFP (see Methods), to determine whether the baculovirus genome is involved in the silencing of gene expression (Figure 3c). Transfected cells treated with butyrate or TSA did not show any significant reactivation of gene expression compared with untreated control cells using this construct, even at the highest butyrate or TSA concentrations tested in this study.

Therefore, all these results demonstrate that transgene expression mediated by recombinant baculovirus containing the human flt-1 promoter is enhanced by the addition of HDAC inhibitors, and the viral genome or proteins coupled to the DNA from the virus are implicated in the silencing of gene expression, since no effect of HDAC inhibitors was observed when the original plasmid used to generate the recombinant baculovirus was directly transfected to drive GFP expression. Moreover, in this study the effect of HDAC inhibitors was independent of the two promoters used.

### In vivo endothelial-specific gene expression by baculovirus vectors containing the human Flt-1 promoter

**Figure 4:**
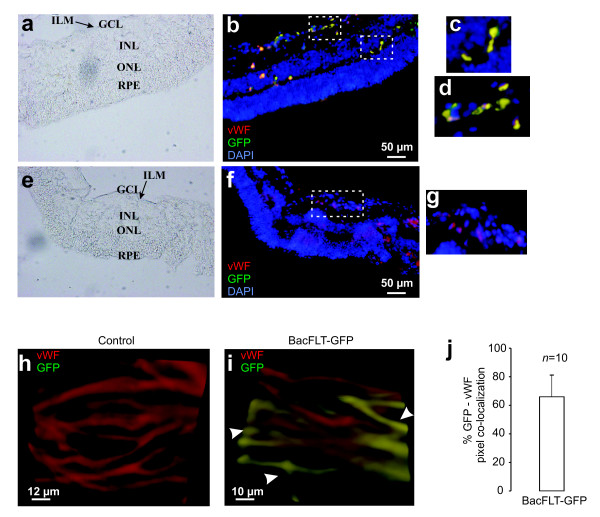
**BacFLT-GFP directs endothelial-specific gene expression into the retina vasculature**. 10 μl of BacFLT-GFP virus solution (approximately 1 × 10^7 ^PFU of viral particles) or vehicle (PBS) were injected into the rat eye vitreous chamber. Three days after the virus injection, the rats were sacrificed and the retinas processed for immunohistochemistry using an antibody against von Willebrand factor (red) and DAPI (blue) to highlight nuclei (see Methods). Representative retinas from eyes injected with 1 × 10^7 ^pfu of BacFLT-GFP (a, b, c, d) or vehicle (PBS) (e, f, g, h). The same sections were evaluated by phase-contrast microscopy (a, e) and by anti-von Willebrand factor antibodies coupled to TRITC (red) and GFP green fluorescence. Merge images illustrate the colocalization of red and green fluorescence (in yellow) in capillary-like structures (big white rectangles in c, d, g) obtained from the areas in small white rectangle insets from b, f. Confocal three-dimensional reconstruction images of retinas injected with vehicle (h) or with BacFLT-GFP (i). In red is shown the localization of von Willebrand factor (red) within characteristic blood vessel structures. The colocalization of red (vWF) and green (GFP) fluorescence is shown in yellow (white arrowheads in i). (j) Percentage of vWF and GFP pixel co-localization in retinas injected with BacFLT-GFP, (*n *= 10 animals). Abbreviations: RPE, retinal pigment epithelium GCL, ganglion cell layer; INL, inner nuclear layer; and ONL, outer nuclear layer; ILM inner limiting membrane.

In order to determine whether the endothelial-specific gene expression mediated by BacFLT-GFP is retained *in vivo*, we selected the eye as a target organ for gene delivery because it is a closed system clearly separated from the systemic circulation, facilitating the delivery of the vector. Furthermore, the blood retinal barrier (BRB) separates the retina from blood, which contains inhibitory factors (e.g. complement) [27]. These characteristic is particularly relevant to baculovirus gene transfer, since complement has been clearly implicated in the inactivation of *in vivo *applied recombinant baculovirus [28]. Moreover, transgene expression in rat retina after intravitreal delivery of recombinant baculovirus has been previously demonstrated, showing that viral particles are able to diffuse through the vitreous body reaching the retina, with maximal GFP expression 2–3 days after injection [29,30]. Based on these considerations, ten microliters of virus (BacFLT-GFP) solution concentrated by ultracentrifugation [approximately 1 × 10^7 ^plaque forming units (PFU) of viral particles] or vehicle as a control were injected into the vitreous cavity of rat eyes. Three days after virus injection, the rats were sacrificed and the retinas were quickly dissected, fixed, and analyzed by fluorescence microscopy using a GFP filter set. Intravitreal delivery of viral particles resulted in strong reporter gene expression, with most GFP-expressing cells almost exclusively localized in the inner limiting membrane (ILM) and ganglion cell layer (GCL). In order to successfully identify the phenotype of the GFP+ cells, we performed immunohistochemical staining showing that most GFP-expressing cells (70%, *n *= 10) react with antibodies to the endothelial specific marker vWF [31], (Figure 4b,c,d,f) confirming the endothelial phenotype of the *in vivo *transduced cells. In contrast, control eyes injected with vehicle alone did not show positive signal for GFP (Figure 4f,g).

To further confirm that our baculovirus vector was driving GFP expression in endothelial cells, we performed tridimensional confocal reconstructions of retinal sections from control and BacFLT-GFP treated animals. Figure 4i illustrates tridimensional projections of the confocal reconstructions clearly showing the blood vessel pattern of GFP expression. Blood vessels were not only recognized for their morphology, but also by immunoassaying with a TRITC-coupled anti- vWF antibody (shown in red). Additional file 1 illustrates the three-dimensional reconstructions (rotating vessels) obtained with retinas from mock-injected animals and animals injected with BacFLT-GFP. These data shows the selective labeling of blood vessels, or portions of them with our recombinant baculovirus.

## Discussion

In the present study, we have demonstrated that recombinant baculovirus containing the 1-kb region from the human flt-1 promoter is able to drive transcriptional gene targeting *in vitro *and *in vivo *in the retinal vasculature. The immortalized bovine umbilical vein endothelial cell line BUVEC-E6E7-1 and other non-endothelial cell types (i.e. C6, HepG2, HEK293, RINm5F) were susceptible to BacCMV-GFP vector-mediated gene transfer. Of all cell lines explored, the highest levels of expression were observed with the hepatocarcinoma cell line HepG2, as previously reported [8,9]. In agreement with the results recently published by Wang *et al*., we also demonstrate that rat glioma cell line C6 is highly susceptible to transduction (> 50% GFP+) by this recombinant baculovirus [32].

In addition, our results show that the endothelial cell line BUVEC-E6E7-1 appeared to be more susceptible to transduction than the human carcinoma/endothelial cell-like ECV-304, in which 21% of the cells were transduced at a MOI of 1000. Such differences could be due to different conditions in which the vectors were titered [33].

The susceptibility of transduction demonstrated in RIN-m5F cells, corroborates the results observed by Ma *et al*., in which pancreatic islet cells were efficiently transduced by recombinant baculovirus carrying a construct similar to the one reported here [34].

Reversible acetylation of the histone tails by histone acetylases (HATs)/histone deacetylases (HDACs) is one of the best studied posttranslational modifications of histones, correlating with transcriptional activation/repression. We have shown here that the treatment of cells with butyrate, an histone deacetylase inhibitor enhanced the transcriptional activity mediated by both BacCMV-GFP or BacFLT-GFP. However, histone hyperacetylation is one of many cellular changes induced by butyrate. Therefore, to confirm whether histone acetylation is involved in reactivation of transgene expression, we also tested the potent and selective HDAC inhibitor, TSA. To date 17 human genes have been identified encoding HDACs, 11 of these genes, members of so-called clase I and II, are inhibited by TSA [35]. Treatment with TSA reactivates GFP expression mediated by BacFLT-GFP at concentrations as low as 10 nM, and maximal effects were observed at 50 nM, corresponding to a concentration 10,000 fold smaller than the concentration of butyrate utilized to reach similar effects (81.68-fold and 61.69-fFold for 15 mM butyrate and 50 nM TSA, respectively). To determine whether DNA methylation is also implicated in baculovirus silencing, 5'aza-C, which is known to interfere with the process of DNA methylation, was added to a concentration of 25 μM alone or in combination with butyrate or TSA. We did not observe a significant increase of transgene expression with 5'aza-C (data not shown). Therefore, DNA methylation does not appear to play an important role in baculovirus-mediated gene transfer silencing. In contrast, histone deacetylation appears to be a general mechanism for silencing baculovirus independently of the promoter or the gene utilized (we have observed reactivation of beta-galactosidase with butyrate or trichostatin A as well, data not shown).

Therefore our results suggest an association between the episomal baculovirus DNA and mammalian histones to resemble nucleosomes. A recently published work demonstrates the association between the episomal adeno-associated virus (AAV) vector genome and the acetylated histone H3 [36]. Thus, a similar mechanism may direct the promoter and transgene-independent silencing of recombinant baculovirus observed here.

Although we do not know the exact mechanism(s) by which TSA or butyrate reactivates transgene expression, it is possible that nuclear spatial positioning can influence the levels of expression observed in the mammalian cells transduced by BacCMV-GFP or BacFLT-GFP. Repositioning of baculovirus genome after HDACs inhibition is something we are currently exploring.

A number of reports have shown that viral sequences including viral regulatory elements can interfere with heterologous promoters used to drive transgene expression and may impair tissue-specific or inducible transgene expression [37-39]. Our data demonstrate that the restricted expression in endothelial cells mediated by the flt-1 promoter is not affected by the context of the baculovirus genome or by the mechanisms of silencing, since BacFLT-GFP evoked the highest levels of expression in BUVEC-E6E7-1 (112 ± 6.2% relative to BacCMV-GFP, *n *= 4), compared to the other cell lines. Interestingly, we have obtained efficient GFP expression in BUVEC-E6E7-1 using 100 MOI of BacFLT-GFP, and previous studies have shown that endothelial cells transduced with recombinant adenovirus required MOI of 500 to achieve similar levels of expression in HUVEC and HSVEC [24].

We took advantage of intravitreous injection, in order to analyze *in vivo *endothelial-specific gene expression mediated by BacFLT-GFP into the retinal vasculature. Our results show that the majority of GFP+ cells were found at the inner limiting membrane (ILM) and ganglion cell layer (GCL), a somewhat expected result since these two structures are in closer apposition to the area of injection (the vitreous). Consistent with this finding, VEGFR1 and VEGFR2 mRNA and Flt-1 protein have been localized to the inner nuclear and in the ganglion cell layer of the rat retina [40,41].

By immunohistochemical staining with the von Willebrand factor (vWF), we demonstrated by fluorescence microscopy and three-dimentional confocal analyses that most of the GFP positive cells are labeled with this endothelial cell specific marker, and localize in blood vessels, showing the specificity of gene expression *in vivo*.

We have observed direct GFP fluorescence in retina slices transduced with BacFLT-GFP even in the absence of histone deacetylase inhibitors; this is probably due to the strong expression driven by the human Flt-1 promoter, which can overcome the silencing effect of histone deacetylases.

Transgene expression in retina after intravitreous body injection of recombinant baculovirus has been previously documented [29]. In this study, a recombinant baculovirus carrying GFP under the control of CMV promoter was subretinally injected, resulting in widespread transgene expression in the corneal endothelium, lens, the retinal inner nuclear layer, GCL, and retinal pigment epithelial (RPE). Unlike the previously mentioned study, the endothelium-selective expression observed in our results originates from the use of the human flt-1 promoter.

## Conclusion

In summary, our study indicates that specific gene expression in the vascular endothelium mediated by the human flt-1 promoter is retained in the context of the baculovirus genome. However, the reactivation of transgene expression by histone deacetylase inhibitors such as TSA or butyrate, suggest that baculovirus genome forms a chromatin-like structure assembled into nucleosomes after the viral genome is delivered into mammalian cells. Future experiments will address the nature of the proteins that affect the silencing of baculovirus vectors in mammalian cells. Finally, this study provides the proof of principle of baculovirus mediated gene transfer to endothelial cells *in vivo *and suggest the possibility of using this recombinant baculovirus for targeted gene expression into the retinal vasculature.

## Methods

### Construction of transfer plasmids

pBlueCMV was generated as follows, briefly a *Hin*dIII-*Sma*I 1.1 kb fragment from pcDNA 3.1(+) (Invitrogen, San Diego, CA) containing the polyadenylation sequence of the Bovine Growth Hormone was inserted into *Hin*dIII-*Sma*I digested pBlueScript (+) (Stratagene, La Jolla, CA) to produce pBSpolyA. A fragment *EcoR*I-*BamH*I from pBSpolyA was cloned in *Eco*RI-*Bgl*II sites of pBlueBac 4.0 (Invitrogene, San Diego, CA) in the opposite orientation to the polyhedrin promoter to produce pBB4polyA. Finally, a *Sal*I-*Eco*RI 953 bp fragment which contains the CMV was ligated into *Sal*I-*EcoR*I sites of pBB4polyA to generate pBlueCMV. This plasmid was designed to contain a multiple cloning site to insert any cDNA under the control CMV promoter. The transfer plasmid pBlueCMV-GFP was constructed by ligation of a fragment *Bam*HI-*Not*I 730 pb containing the coding region GFP from pEGFP-N1 (Clontech, Palo Alto, CA) into the *Kpn*I-*Apa*I sites of the pBlueCMV by blunt end ligation. The transfer plasmid pBlueFLT-GFP was constructed by cloning a *BamH*I-*Hind*III 1.0 kb fragment containing the human *fms*-like tyrosine kinase-1 promoter (hFLT-1 promoter, segment extending from -748 to +284) from the plasmid p(-748/+284) [16] kindly provided by Dr. Andrew Baker (University of Glasgow, Glasgow, UK) into *Bgl*II-*Hin*dIII sites of pBlueCMV-GFP. The correct sequence of each transfer plasmids were confirmed by DNA sequencing.

### Production of recombinant baculovirus

The recombinant baculovirus BacCMV-GFP and BacFLT-GFP, were generated by using the Bac-N-Blue Transfection Kit according to the manufacturer's instructions (Invitrogen, Carlsbad, CA) and were plate-purified twice before to large-scale production. To propagate the baculovirus Sf9 insect cells were infected (2 × 10^6^/ml) at a multiplicity of infection of 0.1 and the viruses were purified as follows; culture supernatants were harvested at 6 days after infection and cells debris was removed by centrifugation at 6,000 g for 15 min at 4°C. The supernatants obtained were titered by plaque assay on Sf9 insect cells, stored at 4°C and used for *in vitro *experiments. In the experiments for gene transfer *in vivo*, recombinant baculovirus were concentrated by ultracentrifugation in a SW28 rotor (Beckman) at 27,000 rpm for 60 min, resuspended in phosphate-buffered saline (PBS) and loaded on 10–50% (wt/vol) sucrose gradients, and was ultracentrifuged at 27,000 rpm for 60 min. The virus band was colleted and diluted in PBS and was untracentrifuged at 27,000 rpm for 150 min in SW28 rotor. The virus pellet was resuspended in PBS and titers were determined as above described.

### Cell culture and transduction with recombinant baculoviruses

Insect Sf9 (*Spodoptera frugiperda*) cells were obtained from Invitrogen (San Diego, CA) and were grown in Grace's media (Sigma, St. Louis, MO) containing 10% (vol/vol) of heat-inactivated fetal bovine serum (FBS) (Invitrogen, Grand Island, NY), 1.0% of lactalbumin hydrolysate, 1.0% yeastolate, penicillin (100 U/ml), streptomycin (100 U/ml) and 0.1% (vol/vol) pluronic F-68 (Invitrogen, Grand Island, NY). Mammalian cell lines including human (HepG2, HEK-293) and the rat cell lines (C6, RIN-m5F) were purchased from American Type Culture Collection (ATCC). The immortalized bovine umbilical vein endothelial cell line BUVEC-E6E7-1, was grown as previously reported [17]. The human glioblastoma cell line CH235, was provided by A. Gutierrez-Lopez (Instituto Nacional de Rehabilitacion, Mexico, D.F.). All cells were maintained in Dulbecco's modified Eagle's medium (DMEM, Sigma, St Louis, MO), while RIN-m5F were maintained in RPMI 1640 medium (Invitrogen, Carlsbad, CA). All cultures were supplemented with 10% of heat-inactivated fetal bovine serum (FBS) (Invitrogen, Carlsbad, CA), glutamine 2 mM, 1% of antibiotics and incubated at 37°C with 5% CO_2 _in a humidity controlled incubator (NUAIRE, Plymouth, MN). Different cell lines were treated with recombinant baculovirus under similar conditions. The day before transduction, cells were seeded at 1 × 10^5 ^per well in six-well plates under conditions above described. The next day the medium was removed, replaced with virus inoculum diluted in Opti-MEM medium (Invitrogen, Carlsbad, CA) to yield multiplicities of infection indicated in the figure legends and incubated during 4 h at 37°C. The inoculum was then replaced by 2 ml of fresh medium with or without sodium butyrate or trichostatin A (TSA) (Sigma, St. Louis, MO) at different concentrations above indicated. Cells were analyzed for GFP expression by fluorescence microscopy and Fluorescence activated cell sorting (FACS) was performed 48 h post-transduction.

### Transient transfection experiments

BUVEC-E6E7-1 cells (2.5 × 10^5^) were transfected with 2.5 μg of the transfer plasmid pBlueFLT-GFP (see construction of transfer plasmids), using Lipofectamine plus (Invitrogen, Carlsbad, CA) according to the manufacturer's instructions. After transfection cells were treated with different concentrations of sodium butyrate or TSA and the levels of GFP expression were analyzed 48 h after transfection by FACS.

### Fluorescence-activated Cell Sorting

The percentage of transduced (GFP+) and mean fluorescence intensity (MFI) was assessed by flow cytometry (FACSCalibur, BD Biosciences). Untreated cells were used to adjust the number of GFP+ cells and mean fluorescence intensity (10,000 events/sample). Acquisition and analysis of FACS data were performed using CellQuest software (BD Biosciences, Palo Alto, CA).

### Animals

Animal care and treatment were according to the ARVO "Statement for the Use of Animals in Ophthalmic and Vision Research." Male Wistar rats (200–250 g) were anesthetized with 70% ketamine/30% xylazine (1 μl/g body weight i.p.) for intravitreal injection of recombinant baculovirus.

### In vivo Gene Transfer

For *in vivo *viral delivery, rats were anesthetized, and their eyes were perforated with a 29-gauge needle to insert a microsyringe (Hamilton, Reno, NV). 10 μl of the vitreous body were extracted, immediately after this 10 μl (approximately 1 × 10^7 ^PFU of viral particles) of recombinant baculovirus or vehicle (PBS) were injected into the vitreous cavity.

### Immunohistochemistry

Three days after the virus injection, the rats were sacrificed and the retinas were quickly dissected and fixed in 4% paraformaldehyde-PBS (pH = 7.4) for 6 hours and then placed in 10% sucrose for 3 hours, 20% sucrose for overnight, and 30% sucrose for 3 days. The tissue was then embedded in optimal cutting temperature (OCT) compound (Tissue-Tek; Sakura Finetek, Torrance, CA), and sectioned at 12 μm. Retinal sections were blocked (0.05% Triton X-100, 1% BSA and 1% NGS) and washed with PBS (pH 7.4). Next, the sections were incubated overnight with an antibody against von Willebrand factor (ZYMED Laboratories, South San Francisco, CA) which specifically stains endothelial cells. Retinal sections were washed and incubated with a rabbit anti-goat TRITC conjugated antibody (Sigma-Aldrich USA, 1:100) and 4',6-diamino-2-phenylindole (DAPI; Vector Labs) was added to label nuclei. Immunofluorescence labeling was observed under a microscope equipped with fluorescence illumination (model BX60; Olympus, Lake Success, NY).)

### Confocal microscopy and image deconvolution

Confocal experiments were performed in a FV1000 (Olympus, Japan). GFP fluorescence was obtained after exciting the samples with 488 nm and reading the fluorescence at 540 nm. For the detection of TRITC the excitation wavelength was 554 and emission was collected at 576 nm. Images were digitized and evaluated with Image Pro-Plus 5.1 software (Media Cybernetics, San Diego, CA). Blood vessel reconstruction was achieved by performing optical slices of retina mounts taken 0.3 μm apart in stacks of about 30 images. All images were obtained from a minimum of 2 slices from at least 2 different animals.

Image stacks of 30 images obtained at 0.3 μm intervals were deconvolved using Autodeblur-X-G-CF software (Media Cybernetics, Inc. Silver Spring, MD). All images were background subtracted prior to deconvolution. Three-dimensional projections were performed with ImagePro Plus v6 (Media Cybernetics, Inc. Silver Spring, MD). Movies showing rotating images were produced with Adobe Premier Pro CS3 (Adobe Systems Incorporated, San Jose, CA).

### Statistical analysis

All data were analyzed using the MedCalc Software (Frank Schoonjans, Belgium) by unpaired Student's *t*-test and are shown as mean ± standard deviation (SD). Data were considered significant when *P *< 0.05. All experiments were performed in triplicate and repeated on at least three independent occasions.

## Competing interests

The author(s) declare that they have no competing interests.

## Authors' contributions

LMA carried out the production of recombinant baculovirus, the experiments *in vitro*, immunohistochemistry and drafted the manuscript. CC designed and supervised the *in vivo *studies and data analyses. JA carried out the virus injection and data analyses. LV conceived the study and designed several of the experiments and complete the manuscript. All authors read and approved the final manuscript.

## Supplementary Material

Additional file 1 Three-dimensional reconstructions (rotating vessels) from retinas obtained from mock-injected animals and animals injected with BacFLT-GFP. A movie showing the rotating vessels obtained from mock-injected retinas and transduced with recombinant baculovirus. Images collected with a confocal microscope and deconvolved to reduce optical aberrations and out of focus fluorescence (see Methods). Green shows GFP expression, red von Willenbrand factor and in yellow is illustrated the co-localization of both fluorescent markers. Click here for file
